# Pathological Outcomes Following Radical Prostatectomy for Low-Risk Prostate Cancer

**DOI:** 10.7759/cureus.91854

**Published:** 2025-09-08

**Authors:** Kayna Fichadia, Aryan Kapur, Cheryl Fung, Basil Razi, Henry H Woo

**Affiliations:** 1 Blacktown Mount Druitt Clinical School, Western Sydney University, Blacktown, AUS; 2 Department of Urology, Blacktown Hospital, Blacktown, AUS

**Keywords:** active surveillance, prostate cancer, prostate mri, psma pet/ct, radical prostatectomy

## Abstract

Introduction

There is an increasing view that the standard of care for men diagnosed with low-risk prostate cancer (LRPC) should be conservative, whether it be with active surveillance (AS) or watchful waiting. The introduction of imaging such as prostate magnetic resonance imaging (MRI) and prostate-specific membrane antigen positron emission tomography/computed tomography (PSMA PET/CT) has provided additional tools to predict the likelihood of occult clinically significant prostate cancer (PC) prior to biopsy and may also play a role in predicting missed clinically significant prostate cancer when prostate biopsies are either negative or only find low-grade PC. The New South Wales Prostate Cancer Outcomes Registry (NSW-PCOR) lists radical prostatectomy (RP) for LRPC as a performance metric for participant urologists, to whom such data is provided through Quality Reports. Given that factors beyond a diagnosis of LRPC may influence a decision for RP, a high level of pathological upgrade is anticipated in men with LRPC undergoing surgery in contemporary practice. The aim of this study was to evaluate the final pathological outcomes of men with LRPC who had undergone an RP and what factors played a role in the decision to undergo surgery.

Methods

The NSW-PCOR Quality Reports were analyzed from inception, and 10 patients who had undergone RP for LRPC were identified. Data, including biopsy histopathology, RP histopathology, and indications for RP, were extracted from clinical records. Clinically significant PC was defined as a Gleason score of ≤3 + 4 or the presence of extraprostatic extension or seminal vesicle invasion.

Results

It was found that seven out of 10 men had clinically significant PC on their RP histopathology. For eight men, preoperative imaging with prostate MRI or PSMA PET/CT had suggested a mismatch between imaging and biopsy pathology of low-grade PC. Of these eight men, seven were found to have clinically significant PC on RP. Other reasons for RP being performed included young age at diagnosis and the request for intervention related to patient anxiety.

Conclusion

In this contemporary cohort of men undergoing RP for LRPC, clinically significant disease was correctly predicted for all but one, where imaging had prompted intervention. Caution needs to be applied both in instances of reliance on imaging to predict occult clinically significant PC, as well as in the appropriateness of assigning conservative management purely on the basis of PC risk stratification.

## Introduction

Prostate cancer (PC) is currently the most commonly diagnosed malignancy and the second-most common cause of cancer-related death in men in most countries [1]. Of these PC diagnoses, low-risk prostate cancer (LRPC) accounts for 45% of newly diagnosed PCs [2]. Currently, the use of active surveillance (AS) for LRPC is a performance metric within the Australian and New Zealand Prostate Cancer Outcomes Registry (ANZ-PCOR), with the number of patients who should be treated with active treatment methods (such as radical prostatectomy (RP)) being zero [3]. Treatments such as RP have the potential to be associated with unnecessary overtreatment and side effects [4].

According to the National Comprehensive Cancer Network (NCCN) Guidelines for Prostate Cancer, LRPC is defined as having a prostate-specific antigen (PSA) level of ≤10 ng/mL, biopsy Gleason score of ≤3 + 3, and clinical stage of ≤T1-2a [5]. LRPC is widely considered to be clinically insignificant and, for the great majority of cases, is most appropriately managed conservatively. Conservative management, either with AS or watchful waiting, remains the initial treatment method of LRPC, being linked to a long-term survival rate of 95-100% [1] and being chosen by over 50% of those with LRPC [6].

There has been a steady decrease in the percentage of men with LRPC undergoing RP, with this number decreasing from 39.6% in 2005 to 9.4% in 2019 [7]. Accordingly, the number of men choosing AS has been trending upward, with there being a 90% increase in a 6.5-year period, as found by a 2023 study [8]. The decision to continue AS has been shown to be influenced by a number of factors, most notably by suggestion from the patient’s urologist [6], with factors like emotional distress being associated with choosing aggressive treatment methods, including radical prostatectomies [9].

There has been evidence that imaging with both prostate magnetic resonance imaging (MRI) and prostate-specific membrane antigen positron emission tomography/computed tomography (PSMA PET/CT) provides an accurate assessment of the clinical significance of intra-gland PC [10]. In particular, prostate MRI is increasingly used as a strategy to reduce the need for repeated prostate biopsies for men on AS [11]. The use of imaging can potentially better stratify those men who are harboring clinically significant PC [12].

Given that factors beyond purely NCCN risk stratification parameters are being increasingly considered in planning management for localized PC, the aim of this study was to evaluate the final pathological outcomes of men who have undergone RP for LRPC, as well as the reasons for proceeding with RP. 

## Materials and methods

The study is a retrospective cohort study. Men who had undergone an RP for LRPC were identified from surgeon-specific (HHW) Quality Reports provided by the New South Wales Prostate Cancer Outcomes Registry (NSW-PCOR). The NSW-PCOR is a state-based contributor to the overall ANZ-PCOR and has an independent governance structure. Each participant surgeon is provided with regular Quality Reports on at least a twice-yearly basis. HHW has been a participant in the NSW-PCOR since its inception in 2015.

As RP for LRPC is a quality metric in the NSW-PCOR, a specific section outlining the names of patients who have undergone an RP for LRPC provides the basis for subjects included in this study. A specific subsection of the Quality Reports was an identifiable list of all patients who had undergone surgery for LRPC. Those patients who were identified as having undergone RP for LRPC from the Quality Reports provided for HHW were selected for this study. These patients formed the inclusion criteria for the study, and no exclusions were made from this NSW-PCOR-defined list. 

The names of selected patients were searched in private practice medical software (Genie Practice Management Software) and clinical details extracted. Patient information was then entered into an MS Excel (Microsoft Corporation, Redmond, Washington, United States) spreadsheet in a de-identified manner. 

Information collected included age at time of diagnosis, biopsy histopathology, imaging results, surgery histopathology, and the reasons behind proceeding with RP for each patient. Biopsy histopathology included primary and secondary Gleason scores, presurgery PSA level, and cT stages. Imaging examined included multiparametric MRI and PSMA PET/CT from which the Prostate Imaging Reporting and Data System (PI-RADS) score, MRI changes, PSMA SUVmax, and PRIMARY score [10] were recorded. Surgery histopathology recorded included primary and secondary Gleason grade, the presence of extraprostatic extension and seminal vesical invasion, and surgical margin status. Additional pathological information extracted included the volume of the dominant nodule, the index lesion percentage, the Gleason grade of 4/5, and the pathological T stage. Insights into the reasons behind RP were derived from the clinical records.

Particular attention was drawn to the comparison of the biopsy histopathology to RP histopathology. Additionally, evaluation of imaging was undertaken to determine its role in identifying disease progression or the presence of a mismatch between imaging results and pathology. Factors that might be suggestive of clinically significant PC on RP pathology were also evaluated. 

We defined clinically PC as being cancer that had a Gleason score of >3 + 4 or had the presence of local invasion into extraprostatic tissue or seminal vesicle invasion. This study was approved by the Western Sydney Local Health District Human Research Ethics Committee (HREC: 2025/ETH01021).

## Results

**Figure 1:**
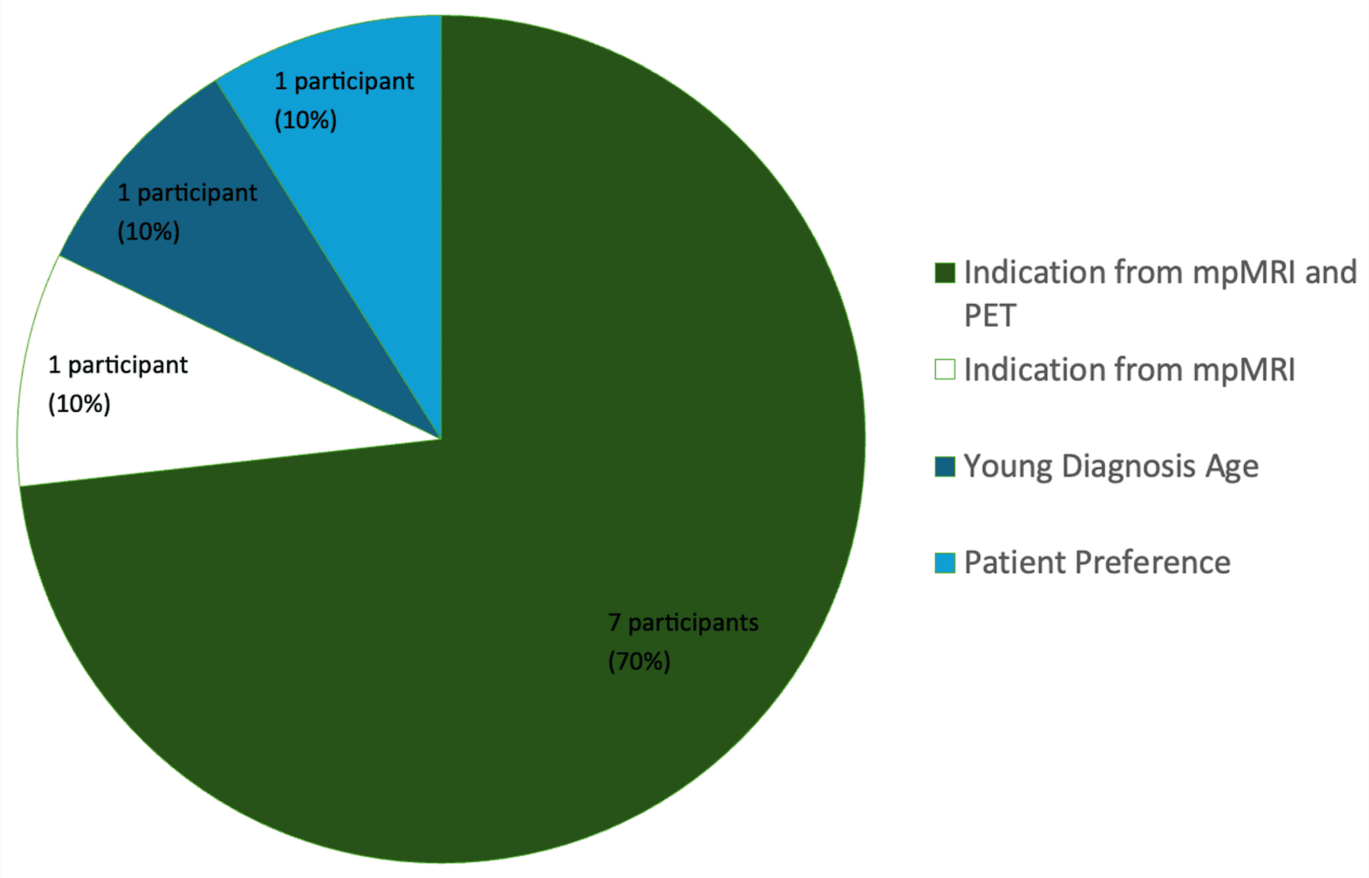
Reason for undergoing RP RP: radical prostatectomy

From the 10 patients identified by NSW-PCOR Clinician Quality Reports, each was confirmed to have met the criteria of LRPC as defined by a PSA level of ≤10 ng/mL, biopsy Gleason score of ≤3 + 3, and ≤cT1-2a. Furthermore, each had undergone RP for reasons including personal preference, young age of diagnosis, or advanced pathology suggested by mpMRI or PSMA PET (Figure 1). For two patients, any potential mismatch between PSMA PET/CT and biopsy could not be deduced on the basis that they did not undergo PSMA PET/CT scans. All patients included in the study had a Gleason score of 3 + 3 and all cT (clinical) stage of T1c. Further, eight of the 10 presurgery PSA levels were between 3.2 ng/mL and 4.6 ng/mL, with the remaining being 6.7 ng/mL and 6.9 ng/mL.

**Figure 2:**
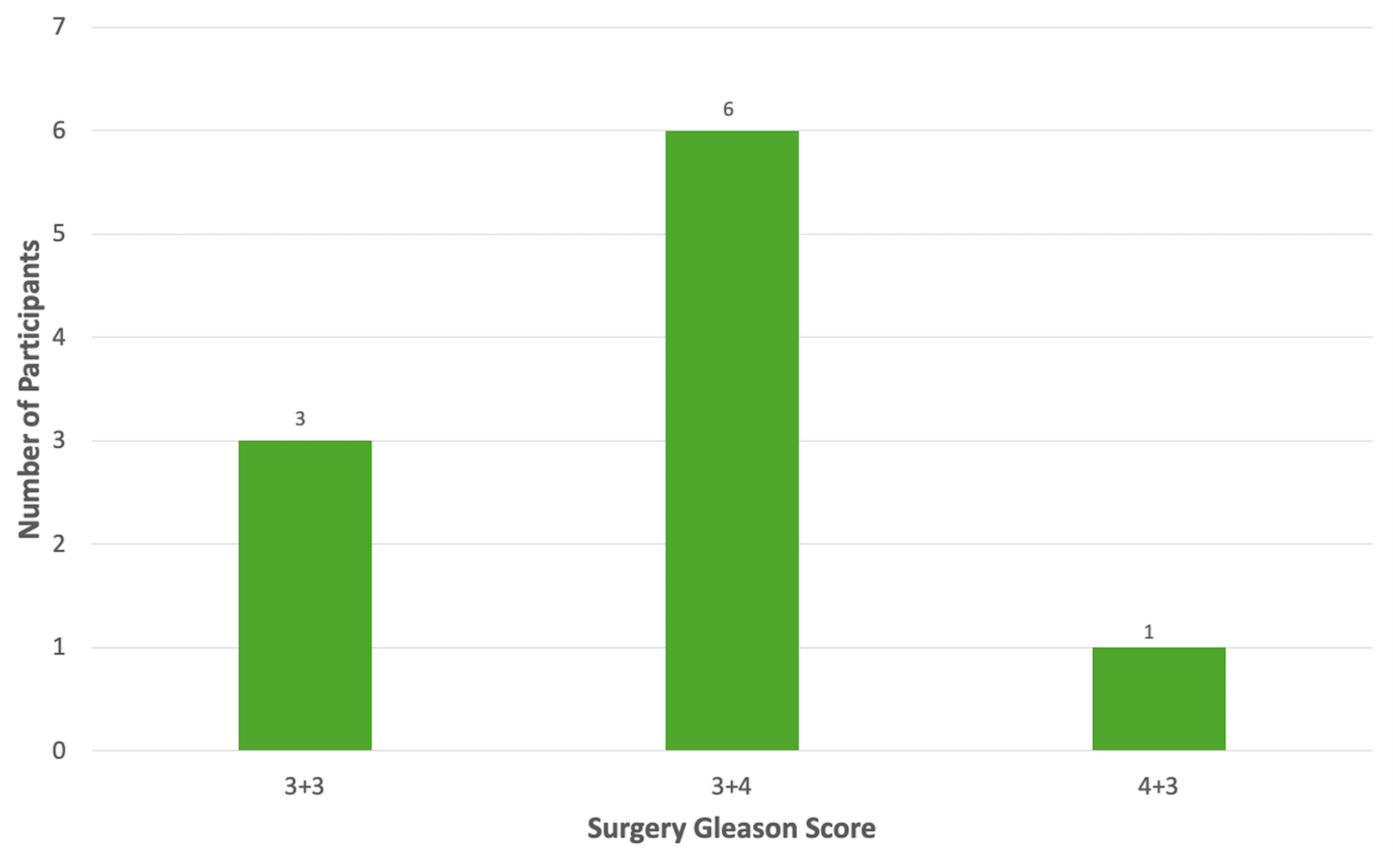
Surgery Gleason score

Of all patients included in this study, seven were found to have clinically significant PC following RP. Six patients had a Gleason score of 3 + 4, and one had a Gleason score of 4 + 3 (Figure 2). Two patients had extraprostatic extension in addition to the upgrading of their Gleason scores. No subjects had evidence of seminal vesicle invasion. No subjects with a Gleason score of 3 + 3 cancer on their final pathology had evidence of extraprostatic extension or seminal vesicle invasion.

Impact of imaging (multiparametric MRI, PSMA PET/CT, or both)

A total of eight patients were recommended RP based on imaging results, being either both mpMRI and PSMA PET/CT or solely mpMRI. Seven patients were recommended RP based on both their mpMRI and PSMA PET/CT scans, which suggested worse pathology despite stratification as LRPC. These patients all had a PSMA PET/CT PRIMARY score of 4 and demonstrated a mismatch between their MRI and biopsy, and PSMA PET/CT and biopsy. One of these seven patients had a PI-RADS of 4 on mpMRI prior to RP and was subsequently found to have clinically significant PC with a Gleason score of 3 + 4 and pathological stage T2 (HW1). Four of these seven patients who had a PI-RADS of 5 prior to RP were seen to have clinically significant PC with RP Gleason scores of 3 + 4 and pathological stage pT2.

One patient with a PI-RADS of 5 prior to RP did not have clinically significant PC with a RP Gleason score of 3 + 3 and pathological stage pT2. One patient with a PI-RADS of 5 prior to RP had clinically significant PC with RP Gleason score of 4 + 3 and pT state T3a, as well as extensive extraprostatic extension. 

**Figure 3:**
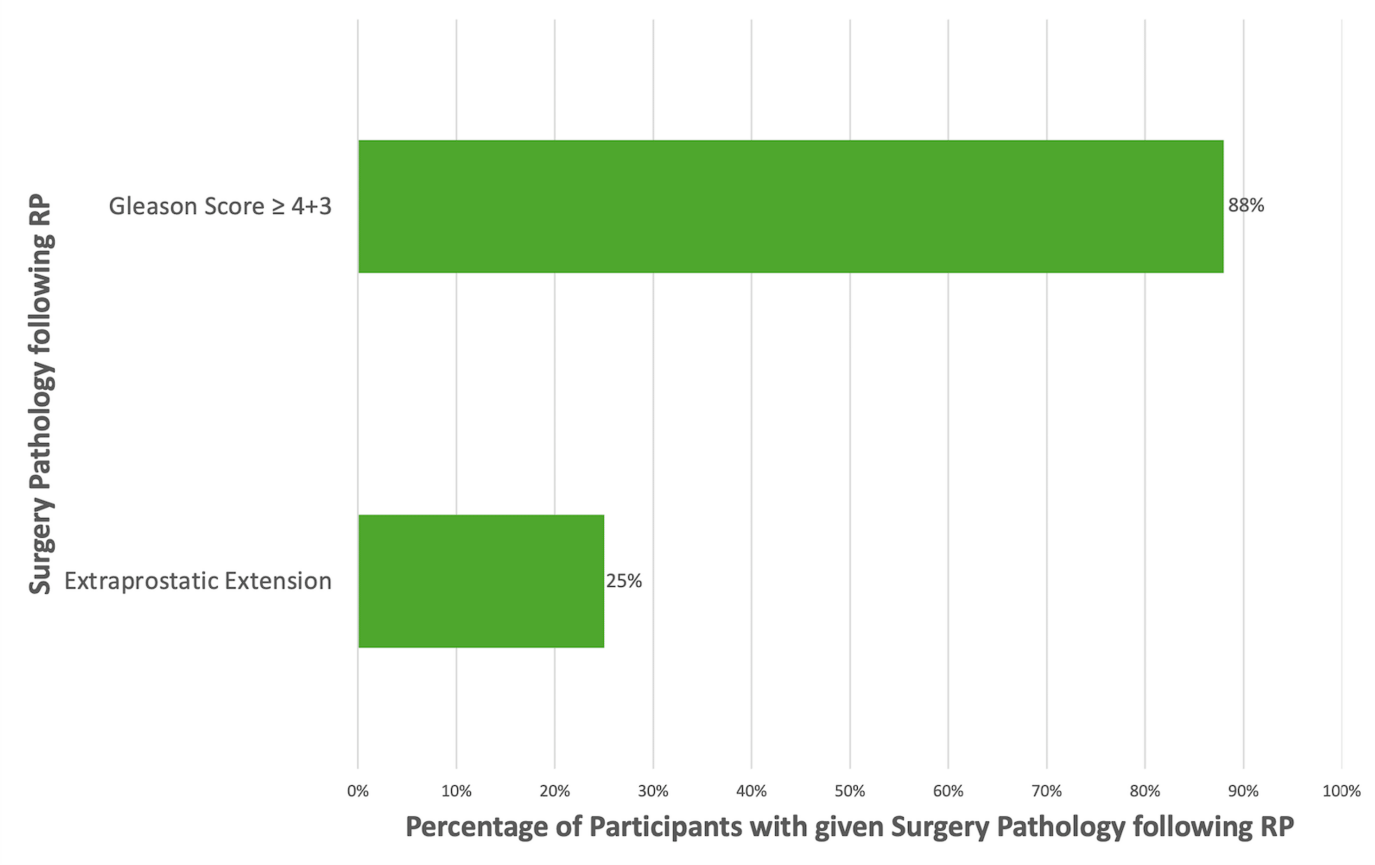
Surgery pathology when RP was indicated by imaging

One patient was recommended RP based solely on a mismatch between MRI and biopsy, having had a PI-RADS of 4 on biopsy. For this patient, a PSMA PET scan had not been done, and resultingly, any potential mismatch between biopsy and PSMA PET could not be determined. Following RP, this patient did have clinically significant PC with a Gleason score of 4 + 3 and pathological stage T3a. Of all eight patients recommended RP based on imaging, seven patients had a Gleason score of ≥3 + 4, and two patients had extraprostatic invasion following RP (Figure 3).

Intervention for other reasons

Of the two remaining participants, the reasoning for undergoing RP included young age (43 years) at the time of diagnosis and participant preference despite the recommendation for AS. One patient was recommended to undergo RP based solely on the young age at the time of diagnosis, being 43 years old. This patient did not demonstrate a mismatch between MRI and biopsy pathology, having a PI-RADS of 2 on biopsy, and had not undergone a PSMA PET scan. Further, there was no indication of clinically significant PC following RP. 

One patient was recommended AS as per their biopsy pathology, mpMRI, and PET scan, with there being nothing to suggest more advanced pathology of their LRPC, but voluntarily chose to undergo RP. The reasoning behind this decision was anxiety surrounding the diagnosis. Following RP, this participant had a Gleason score of 3 + 3, with pathological stage T2.

## Discussion

The key finding of this study is that the majority of men who had undergone an RP for LRPC based on indication from imaging were found to have clinically significant PC. At first glance, the performance indicator in the NSW-PCOR Quality Report indicates 10 men having undergone surgery when conservative treatment should have been offered. Our data suggests that there needs to be caution with leveling any criticism for having performed RP for LRPC. 

AS is a strategy to strike the right balance between avoiding overtreatment of clinically insignificant PC and maintaining the window of opportunity to provide curative treatment in the event that the cancer is found to be of clinical significance. AS has been shown thus far to be effective in reducing overtreatment of LRPC as per the Prostate Cancer Research International: Active Surveillance (PRIAS) study. Despite this conclusion, the study clearly highlights the importance of heeding high-risk features in patients with LRPC and encourages monitoring of these [13].

However, the use of AS does not come without disadvantages such as requiring multiple scans and biopsies, risk of psychological toll associated with intense surveillance, and potentially worse outcomes in the case of cancer progression [14]. One patient in our series made a decision to have surgery despite being recommended AS and was found to have only low-grade PC on final pathology. This patient did not have any imaging or pathological risk factors to suggest likely upgrading or upstaging of his cancer, and he could have safely continued on AS. While AS aims to preserve quality of life, in this instance, it would have been associated with erosion of this patient’s quality of life due to anxiety. Despite these circumstances, this patient still appeared on the RP for the LRPC performance indicator list, which is a metric for the NSW-PCOR. 

In one study, it was suggested that RP could increase survival outcomes of patients with LRPC compared to those with no local treatment in men younger than 74 years old [15]. This, however, directly conflicts with the prevailing opinion, as established by the ProTect study, that radical treatment methods such as RP appear to have no significant impact on mortality over a 15-year period [3].

The use of mpMRI has been shown to improve the accuracy of predicting which patients would benefit from AS when used in conjunction with other risk stratification tools such as the Cancer of the Prostate Risk Assessment, Einstein, and D’Amico [16,17]. In spite of this known benefit of mpMRI, the use of mpMRI as a method to indicate advanced underlying pathology in those patients with LRPC is not accepted as justification for RP. 

The ability of individual imaging methods, including mpMRI and PSMA PET scans, in providing an indication of advanced pathology could not be assessed independently of one another in this study due to PSMA PET/CT scans not being performed in all participants. At the time of the study, the use of PSMA PET/CT in Australia was not commonplace or reimbursed by Medicare Australia. Resultantly, the effectiveness of these imaging methods in indicating advanced pathology could not be compared. 

This study is not without limitations. All 10 participants of this study were derived from a single surgeon’s cohort of patients. This small sample size negatively impacts the reliability and generalisability of these results. There were also data points that could not be consistently obtained or assessed, including cribiform architecture and intraductal carcinoma. The presence of cribiform architecture and intraductal carcinoma morphologies has been shown to indicate negative pathological and oncological outcomes [18]. Data points on these would provide indications of poor prognosis among patients, further determining if the factors beyond risk stratification that were used to make the decision to proceed with RP were reliable indicators of underlying advanced pathology. Five years earlier, these high-risk pathological features were not always reported consistently, although it is now routinely reported. 

This data brings into question the reliability of surgery for LRPC being a measure of quality. Further evaluation of a large dataset, as is the case for the NSW-PCOR, is important to demonstrate if the incidence of pathological upgrading as seen in this study is potentially generalizable in contemporary practice. Interrogation of the NSW-PCOR for such data is currently underway. 

## Conclusions

In a contemporary cohort of men undergoing RP for LRPC, clinically significant disease was correctly predicted for all but one, where imaging had prompted intervention. An increasing use of sophisticated imaging, such as prostate MRI and PSMA PET/CT, will likely see these results input into the decision-making process for managing LRPC. Whether a repeat prostate biopsy should always be performed prior to RP is yet to be clarified in a randomized trial. For now, caution needs to be applied when relying on imaging to predict occult clinically significant PC. Additionally, there needs to be caution applied to the interpretation of the performance metric of RP for LRPC. It also raises the question as to the appropriateness of conservative management purely on the basis of prostate risk stratification.
